# Glass–Adhesive–Steel Joint Inspection Using Mechanic and High Frequency Electromagnetic Waves

**DOI:** 10.3390/ma13204648

**Published:** 2020-10-18

**Authors:** Jakub Kowalczyk, Przemyslaw Lopato, Grzegorz Psuj, Dariusz Ulbrich

**Affiliations:** 1 Department of Civil and Transport Engineering, Institute of Machines and Motor Vehicles, Poznan University of Technology, 60-965 Poznan, Poland; jakub.kowalczyk@put.poznan.pl; 2 Department of Electrical and Computer Engineering, West Pomeranian University of Technology, 70-313 Szczecin, Poland; plopato@zut.edu.pl (P.L.); gpsuj@zut.edu.pl (G.P.)

**Keywords:** adhesive joint, ultrasound, terahertz inspection, microwave imaging, thermography

## Abstract

The utilization of a glass–adhesive–steel joint in modern machine and vehicle production is constantly growing. Despite the technological regime during the production process, various defects in the adhesive joints may occur. One of the major problems is lack of adhesive between joined materials. Due to the widespread use of non-transparent layers increasing adhesion, it is frequently not possible to conduct simple visual inspections. Hence, it is important to develop a non-destructive adhesive path continuity examination procedure. In that process, the different physical properties of the joint materials must be taken into account. Therefore, in this paper various testing methods were used, including ultrasound, thermographic and electromagnetic methods operating in the microwave and terahertz frequency range. Different physical phenomena of the methods allowed for examination of the joint in a wide context. In order to verify the information brought by each method, the results were transferred into common coordinate space, compared and finally integrated. Various opinion pooling strategies were conducted to fuse data, which allowed us to emphasize convergent and complementary information on adhesive continuity. The obtained results are promising and constitute the basis for further work on an integrated system for automatic evaluation of a wide range of possible defects in glass–adhesive–steel joints.

## 1. Introduction

Adhesive joints are widely used at the stage of manufacturing modern motor and rail vehicles, because they complement other joining methods, such as spot welding, braze welding, riveting and laser welding. This is primarily due to the possibility of connecting various materials (e.g., glass with steel, steel with aluminum, aluminum with glass, etc.). Other advantages of the adhesive joints used are even distribution of stress throughout the entire joint and no damage to the zinc coating protecting the steel against corrosion. In addition, the adhesive suppresses the occurring vibrations and seals the body panel elements connected to the body shell, e.g., connection of the body panel to the roof structure, windscreen to body shell. The most commonly used adhesives at the stage of manufacturing car bodies and rail bodies are epoxy adhesives, MS polymers (modified silane adhesive), polyurethane adhesives and adhesives with an increased modulus of elasticity to connect glass with steel parts. Assessing the quality of glass–adhesive–steel connections is of increasing importance and therefore requires more and more attention. According to the International Organization of Motor Vehicle Manufacturers OICA (Organisation Internationale des Constructeurs d’Automobiles), each year only the bus market in the world increases by almost 300,000 new vehicles. In each of them there are at least a dozen flat-glass window surfaces jointed into a steel frame. In conjunction with the needs of the railway market, where similar solutions for glass–steel joints occur, the need to develop effective testing procedures for this type of joints is gaining importance.

**Figure 1:**
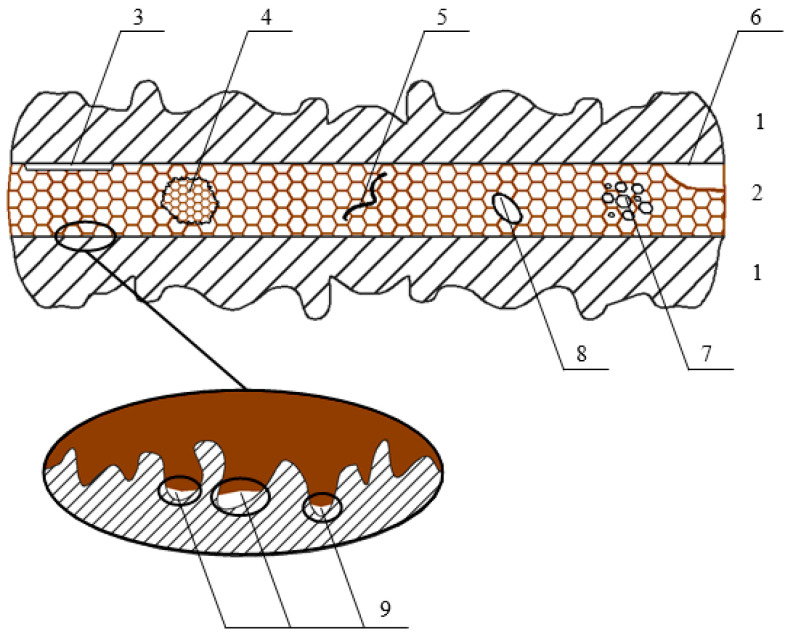
Technological defects of adhesive joints: **1**—joined materials, **2**—adhesive, **3**—de-adhesion, **4**—lack of solidification of the adhesive, **5**—crack, **6**—no adhesive, **7**—porosity, **8**—air bubble, **9**—no fill of irregularities in the surface.

Application of advanced adhesive technologies is not a guarantee of obtaining adhesive joints without defects (especially technological ones) arising at the stage of vehicle production. These include de-adhesion and lack of filling surface irregularities caused by its improper preparation. In addition, a technological flaw is also determined by the partial or complete lack of solidification of the adhesive joint resulting from improperly prepared adhesive or improper solidification conditions, such as, for example, time, temperature and air humidity. An equally important technological defect of the joint is the lack of adhesive in the joined areas resulting from machine or human errors. The same group of defects includes porosity and air bubbles in the joint resulting from incorrect application of adhesive and improper bonding of the materials to be bonded. The basic technological defects of adhesive joints are shown in Figure 1.

**Figure 2:**
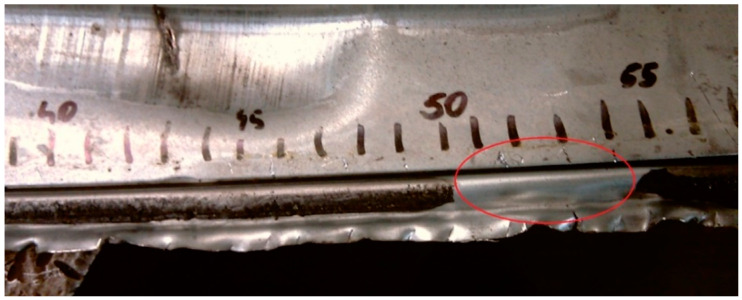
View of example of adhesive joint defects—lack of adhesive path.

A typical defect of adhesive joints of key importance is the lack of adhesive in the area where it should be applied in accordance with the production documentation of the technical object. The problem of lack of adhesive in areas designated for bonding or adhesive path is quite often found in real manufacturing conditions (Figure 2).

It is caused by the influence of structural (improperly designed node shape) or technological (deformation of the adhesive node or errors in the application of adhesive) factors. Mostly, the use of simple visual inspection techniques is not possible. Glass elements are often covered with a non-transparent layer that increases the adhesion of the adhesive, which in consequence limits the possibility of visual assessment. In addition, it should be emphasized that there are large differences in the physical properties of the materials of individual elements that make up the joint. Therefore, it is important to develop and apply non-destructive procedures based on various methods, which allow location of the adhesive path applied between two joined materials. There are a number of methods that can provide relevant data to assist in the evaluation of adhesive joints. It is not always possible to carry out inspections from both sides of the tested component. Therefore, one of the basic criteria for obtaining high efficiency is the ability to use a given technique to obtain data from the full cross-section of a joint while applying it only on one side of the component. However, it should be emphasized that there is no single method to obtain information on all possible aspects of the layered structure being tested. Therefore, it is important to conduct multi-source inspection using a set of methods based on different physical foundations, allowing us to obtain complementary information and creating a more complete set or increasing the certainty of the correct assessment of the tested joint [1,2,3,4]. As a result, such numerous results can then be combined and expressed as a single one to provide a broader spectrum of information on the component state in one form. 

Considering the dielectric properties of the materials (such as glass or adhesive) building the joint, the interest in electromagnetic methods, operating in high frequency bands of the field, increase. One can distinguish here a microwave or especially, recently, a terahertz method. These methods, due to technological progress and decreasing costs of measuring system components, are finding increasing interest of researchers. The observed phenomenon, accompanying the interaction of high-frequency electromagnetic waves, creates opportunities to obtain information from various depths of the material being tested. The group of methods based on the use of high-frequency electromagnetic fields can also be supplemented to a key extent with the infrared thermography technique. The monitoring of infrared emissions of the tested object allows one, above all, to detect existing anomalies in the entire volume of the object. At the same time, using appropriate measurement and image analysis procedures, it is also possible to carry out a more accurate assessment at individual depths in the material. This method is more and more commonly used in many industrial applications. It enables quick inspection at both global (relatively large elements) and local (in selected areas) scales. Those mentioned, high frequency electromagnetic methods are a group of techniques with high development potential. However, they do not belong to the most frequently used in the field of joint testing and still require further development and standardization. In that field, one of the most commonly used measurement techniques is ultrasonic testing, which uses the phenomenon of sound wave propagation in the tested element and response wave observation. Despite widespread use, this technique is under constant development, and new measuring approaches are still being introduced into practice. It also can provide information about the joint structure, while it is related to the differences in speed of sound propagation in the material. The next section will discuss the basic properties of all these research methods.

The microwave, millimeter wave and terahertz nondestructive evaluation methods are based on the same phenomena—high frequency electromagnetic waves. Thus, it can be successfully utilized in adhesive joint inspection. Because of the skin effect, high frequency electromagnetic waves penetrate only surface of conductive material (depending on frequency and electric conductivity, the penetration depth is in the micron range). Therefore, it is necessary that the adhered material of the tested joint from the measuring head side is non-conductive, e.g., polymers, selected composites, wood or glass. Then, the adhesive should be also dielectric or low-conductive, and the second adhered material can be conductive or not—in both cases there will be some adhesive–material boundary reflection pulse. The mentioned techniques differ in frequency range (and as a consequence in spatial and lateral resolution, as well as in the penetration depths) and generation/detection process. Both techniques are not as standardized as ultrasonic or X-ray testing; however, their potential is being developed by many laboratories and applied in industrial inspections. Microwave and terahertz non-destructive evaluation (NDE) techniques were widely utilized for dielectric structure production stage evaluation (including polymerization process monitoring) [5,6], as well as in exploitation stage examination, including aviation, automotive and rail industry structures [7], wind turbine blades [8,9], moisture detection and monitoring [10] and new types of materials examination [1,11,12]. The application of high frequency electromagnetic waves also includes dielectric material based joint inspection—thermal fusion joints (e.g., in the case of HDPE pipes) [13] or adhesive bonds [14]. 

In the case of vehicle components, the thermographic method can be used to control the quality of parts (crack location, delamination, kissing bond) made of carbon fiber reinforced polymer (CFRP) [15,16,17]. The thermographic method is also used in the study of adhesive joints [18]. Nevertheless, in the case of industrial application of this method, the main limitation is the need to change the joint temperature (heating or cooling). It may lead to damage of the joint—adhesive leakage or its detachment from the connected elements. In the further operation of such a joint, this may result in the penetration of water into the vehicle through a leaking adhesive joint. Nevertheless, the method allows relatively rapid inspection, which should stand for a significant advantage. Kurpinski et al. [19] proposed the use of the thermographic method to locate defects in adhesive joints and also to assess the adhesive patch continuity between two steel elements. Other examples of evaluation of adhesive joints using the thermographic method also refer to joining metallic components with adhesive [20,21]. Not only images from a thermographic camera were used in them but also computer simulations. The authors of this publication did not encounter steel–adhesive–glass joints using this method, especially from the glass side.

Ultrasonic testing of adhesive joints has been conducted for many years. These are mainly tests of steel–adhesive bonds [22], aluminum–adhesive bonds [23] and adhesive joints of composite materials [24,25]. Such kinds of research include defect searching in adhesive bonds. Some of the defects found in adhesive joint are kissing bonds [26,27], gaps in the continuity of the applied adhesive path [28] and places in bond with lack of adhesion [29]. Ultrasonic assessment of the fatigue damage of adhesive joints [30] is also one of the research directions conducted by the researchers. Adhesive joints in which glass is one of the joined materials are very rarely examined by the ultrasonic method and described in the literature. Mojskerc et al. [31] attempted the ultrasonic assessment of the joint of hardened glass, polysulfide or silicone adhesive and a polymer profile. They focused primarily on assessing the quality of the bond and defect detection, e.g., air pockets. This type of connection, just like the glass–adhesive–steel joint tested in this article, causes a number of difficulties during ultrasonic measurements due to the specific acoustic impedance of each material. Campione et al. [32] tested the adhesive bond made by two monolithic glass and acrylic adhesive. In this joint, an artificial defect was generated. Ultrasonic impulses from the joint area as well as shear stress–slippage curves from bond tests were obtained.

Considering all the above mentioned aspects, this paper presents a new approach aiming for the verification of the possibility and scope of application of several methods for testing of glass–adhesive–steel joints on a common exemplary sample. Thanks to test procedures proposed in the article, it is possible to compare the results of inspections processed by several methods based on various physical basics (and what is important) obtained under the same conditions. In addition, as part of the work, an attempt was also made to determine and verify the nature of information carried by individual methods in the context of their competitiveness or complementarity. This is important in the appropriate selection of a set of test methods and further in obtaining the desired effectiveness of the measurement system. The mentioned efficiency should be understood as obtaining higher reliability or a broader spectrum of information about the state of the tested element. Therefore, for this purpose, the data obtained by all methods was transformed into a common result space (possibility of compilation) and then combined. Performing data fusion based on logical operators of conjunction and disjunction enables the examination of the scope of convergence and uniqueness of information about the tested joint, which may in the future provide key aspects for the correct assessment of various possible defects. 

## 2. Materials and Methods 

### 2.1. Sample Characteristics

**Figure 3:**
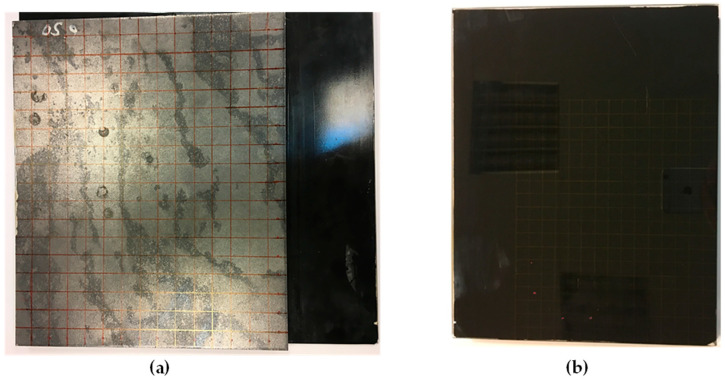
View of both surfaces of sample utilized during experiments: (**a**) view from the steel sheet side, (**b**) view from the side of the glass.

**Figure 4:**
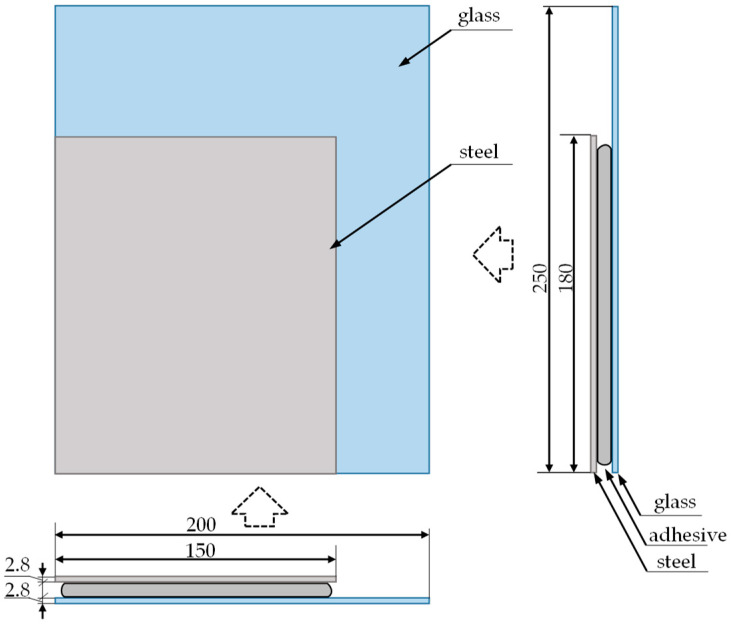
Drawing of the test sample with dimensions (all dimensions are given in mm).

Non-destructive testing was carried out on an exemplary sample (Figure 3 and Figure 4) of a typical joint used in various vehicles, made of structural steel and glass.

Both of these elements were joined with adhesive commonly used in industry (rail and motor vehicle industry). The sample was prepared in accordance with the standards and procedures of the rail vehicle manufacturer (both in terms of material selection and manufacturing technology).

### 2.2. Terahertz Inspection Method

**Figure 5:**
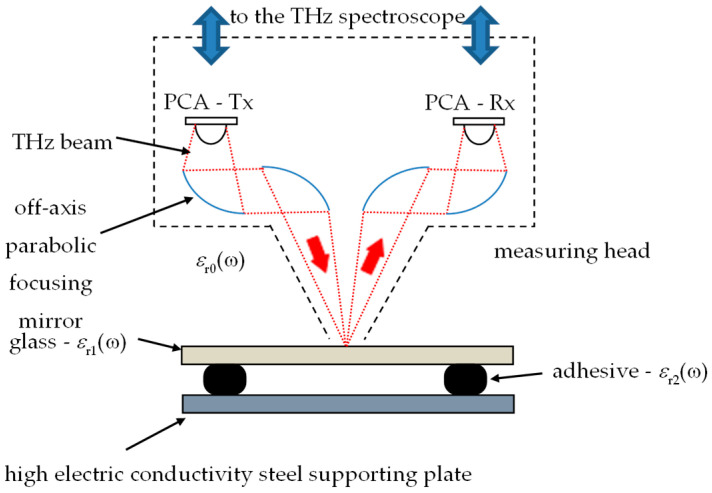
Scheme of measuring setup for terahertz imaging.

**Figure 6:**
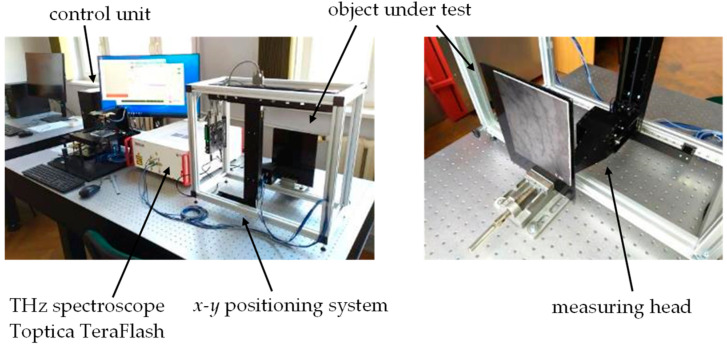
View of terahertz imaging system.

Non-destructive evaluation using high frequency electromagnetic waves (in microwave and terahertz frequency ranges) is based on the measurement of the wavefront reflected off or transmitted through the material under test (MUT). In the case of the transmission setup, the source of electromagnetic field is on the one side of MUT, while the detection/measurement is on the opposite side (mostly utilized in traditional spectroscopic applications). In the case of reflection arrangement (more suitable in most industrial applications), both the transmitter and receiver are on the same side of MUT. This setup was utilized in the presented work, as shown in Figure 5 and Figure 6.

Contrary to ultrasonic testing, the measuring heads (antennas) are not in contact with the evaluated structure, thus no coupling gels or similar techniques are necessary to use. The terahertz method has been used in non-destructive testing of dielectric materials for two decades, which is a relatively short time compared to such methods as radiography or ultrasonic testing. In the case of terahertz imaging, the frequency band of 0.1–10 THz (which corresponds to wavelengths of 3 mm–30 µm in free space, respectively) is considered [5,6,33]. There are several techniques in application, including continuous wave (CW) imaging (single frequency or swept frequency excitation and single point or matrix detection) or time-domain spectroscopy (TDS) with femtosecond order pulsed excitation and detection using photoconductive antennas. The first setup enables higher power (and in consequence testing of thicker and more lossy structures) and faster inspection, while TDS enables spectroscopic information about an examined structure (dispersion curves and absorption peaks for specified frequencies correlated with specific molecules) [7,8,9] and relatively intuitive information about cross-section (in depth profile) because of operating principle similar to radar [10,11,12,25]. The excitation pulse is directed into the examined material and after interaction (reflection of interfaces having various dielectric properties) is picked up by a receiving antenna. The detailed principle of terahertz imaging is presented in [33,34,35,36,37]. In the case of the utilized THz measuring setup, both transmitting and receiving antennas are excited using an optical pulse from a femtosecond laser (part of the THz spectroscope). A few picosecond-long terahertz pulses are generated by the transmitting photoconductive antenna (PCA) and propagate in the form of the beam shaped by the set of off-axis parabolic mirrors. The beam, after passing by two mirrors, is focused. The examined structure is positioned in the vicinity of the focal point. The reflected terahertz beam is further directed (through two additional off-axis parabolic mirrors) to an aperture of a receiving PCA, where induced current is detected. The whole reflection setup is implemented in a measuring head, which is translated over the sample by a two-dimensional positioning system. The photo of the utilized TeraFlash terahertz imaging system (Toptica Photonics A.G., Munich, Germany) is presented in Figure 6. Because of the high electric conductivity of the steel supporting plate, the glass–adhesive–steel joint could be examined only from one side (glass-side, as shown in Figure 5 and Figure 6).

### 2.3. Microwave Inspection Method

**Figure 7:**
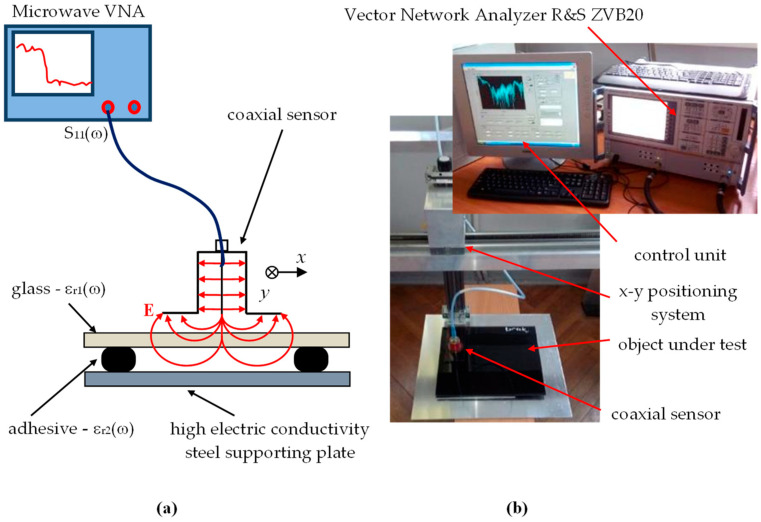
Scheme (**a**) and photo (**b**) of microwave imaging system.

The microwave frequency band is strictly defined as 300 MHz–300 GHz. Compared to the previously described terahertz method, microwave imaging offers lower spatial resolution (caused by longer wavelengths of 1 mm at 300 GHz and 15 mm at 20 GHz in free space) and higher electromagnetic field intensity and penetration depths into lossy materials [38,39,40,41]. Generally, the excitation can be realized similarly to the case of the terahertz technique: CW, swept frequency sinusoidal waveform and pulsed. In the measuring system utilized in this work, sweep of frequency was utilized as a part of the vector network analyzer (VNA) functionality. The scheme and photo of the microwave imaging system based on a ZVB20 VNA (Rohde & Schwarz, Munich, Germany), is presented in Figure 7.

The proposed system and sensor have found successive applications for inspection of dielectric composite materials and structures with nano-fillers [11]. A coaxial sensor is connected to port 1 of the VNA through a 50 Ω coaxial transmission line. The sensor itself can be treated as a coaxial transmission line loaded with variable impedance representing the local properties of the examined structure, which can be also considered as a three-dimensional distribution of dielectric and/or conductive properties. Depending on the dimensions of the coaxial sensor and the excitation frequency, the distribution of the resulting electromagnetic field varies, causing changes of sensitivity and resolution of inspection.

The 10 MHz–20 GHz variable frequency signal generated by port 1 of the VNA is transmitted to the sensor, where it is partly reflected by the examined structure because of differences in intrinsic impedances. Depending on the dielectric property distribution in the vicinity of the measuring transducer/antenna, the measured reflection coefficient S_11_ (ratio between reflected and incident wave) is acquired in complex form (amplitude and phase). The sensor scanned the sample with a step of 2 mm in both (x and y) directions with a liftoff of 2 mm.

### 2.4. Thermographic Method

**Figure 8:**
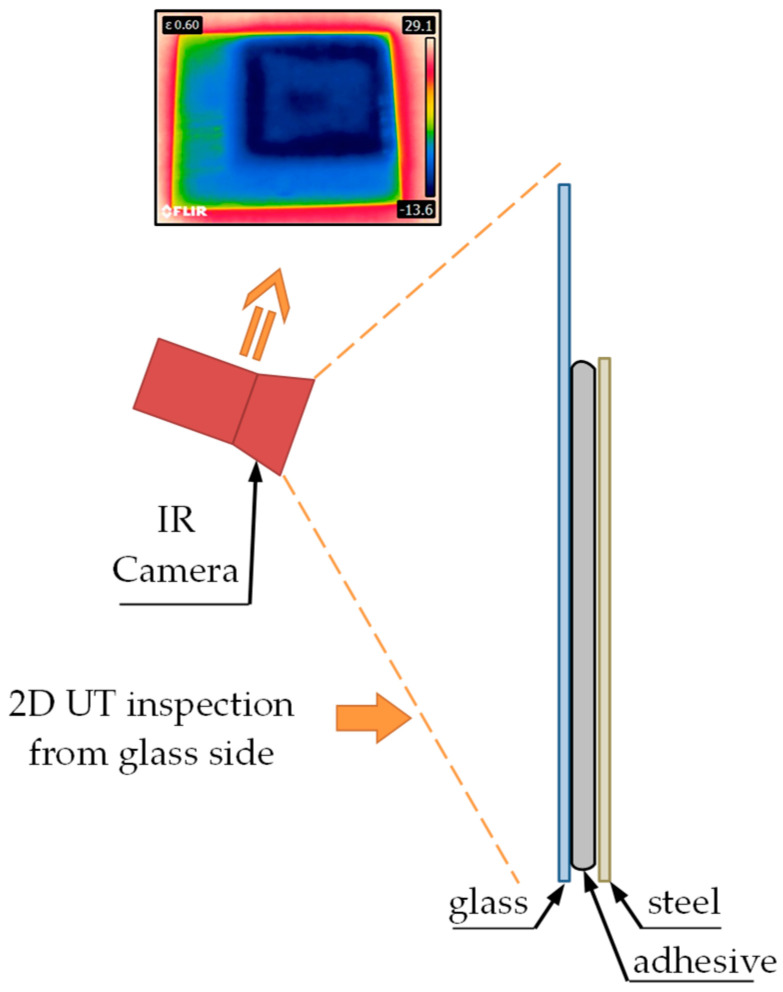
Scheme of the test stand used during the measurements of the sample with the IR camera.

In the case of the thermographic method, the tests were carried out only from the side of the glass, using a Flir e8 infrared (IR) camera (FLIR Systems, Inc., Wilsonville, OR, USA). The detector allows an IR resolution of 320 × 240 pixels to be reached, with maximum acquisition frequency of 9 Hz. Before the acquisition of IR image sequences, the sample was cooled to a temperature of about 3 degrees Celsius in a cooling chamber. The sample was then pulled out and placed on a table in a room at a temperature around 30 degrees Celsius. Locally, the process of equalizing the temperature to the temperature of the external environment depends on the arrangement of materials forming the joint structure in a given place on the measuring sample. The sample has a structure (depending on the location) partly consisting of steel, air and glass, and partly steel, adhesive and glass. Considering the differences in the thermal conductivity of the materials forming the joint, the time necessary to achieve the temperature equilibrium is different and depends on the setup in a given place. Thus, by sequential observation in time, it is possible to distinguish places where there is a layer of adhesive between the steel–glass setup from those locations where there is free air. The steel has the highest thermal conductivity coefficient (approx. 50 W/mK), whereas the glass has a much lower value of the coefficient—close to 1 W/mK. In the case of the adhesive, this value is much below unity, while the lowest coefficient is in the case of air (approx. 0.025 W/mK). High values of thermal conductivity of the steel make it difficult to measure from the steel side. The equilibrium process is relatively rapid on the steel side, and testing of IR emissions from this side requires the use of a fast camera. On the other hand, the much lower dynamics of the process observed on the glass side made it possible to carry out slower IR imaging. Therefore, measurements (photos) of the temperature distribution on the sample were carried out at intervals of 30 s from the moment the sample was placed on the table until there was clear indication of the adhesive in the joint. The adopted time intervals made it possible to observe changes in the temperature distribution on the tested surface, which is discussed in more detail in the next section of the article. The scheme of the test stand used during the test is shown in Figure 8.

### 2.5. Ultrasonic Method

**Figure 9:**
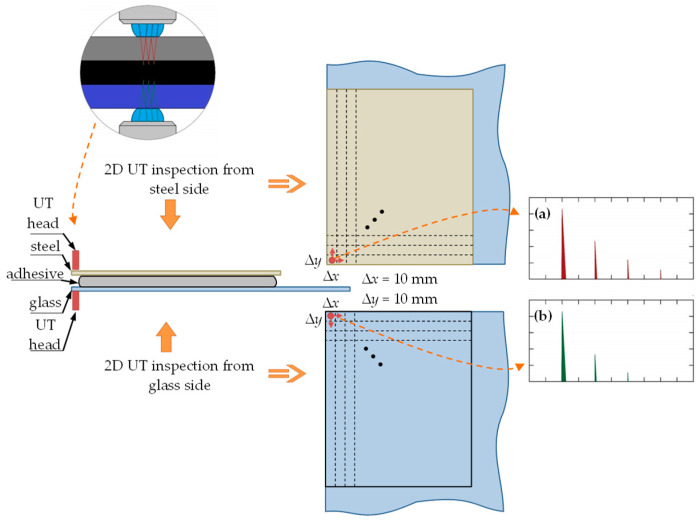
Diagram of ultrasonic testing system and visualization of the wave propagation in the tested sample: (**a**) the steel side testing, (**b**) the glass side testing.

Ultrasonic testing of the glass–adhesive–steel joint was carried out on both the steel and the glass sides. This was due to the strong damping properties of the adhesive. The adhesive caused suppression of the ultrasonic wave penetrating into the adhesive layer. This phenomenon is shown in Figure 9.

Therefore, in the case of the used apparatus, it was not possible to test the entire joint (obtaining longitudinal wave pulses from the bottom of the sample) from one side only.

To define the locations of measurement, registration of selected parameters of the ultrasonic wave and visualization of the results, measuring grids were applied on both the steel and the glass sides. The schematic view of the grid locations is presented in Figure 9.

On each side, the grid with a step of 10 mm in both directions was prepared on a 150 × 180 mm^2^ area. In the center of each area, ultrasonic measurements were made using a 20 MHz ultrasonic head with water delay and the USM 35XS flaw detector (GE, Schenectady, NY, USA). The settings of the ultrasonic apparatus were as follows:

- at the steel side:
  ― type of wave, longitudinal;
  ― ultrasonic wave speed, 5920 m/s;
  ― observation range, 40 mm;
  ― 54 dB ultrasonic wave pulse gain.
- at the glass side:
  ― type of wave, longitudinal;
  ― ultrasonic wave speed, 6000 m/s;
  ― observation range, 40 mm;
  ― 40 dB ultrasonic wave pulse gain.

During the tests, the following parameters of the ultrasonic longitudinal wave were recorded:

- the number of return pulses from the joint area with a constant observation range;
- amplitude of all registered impulses reflected from the connection border (glass–adhesive and steel–adhesive).

## 3. Results and Discussion

### 3.1. Terahertz Imaging

**Figure 10:**
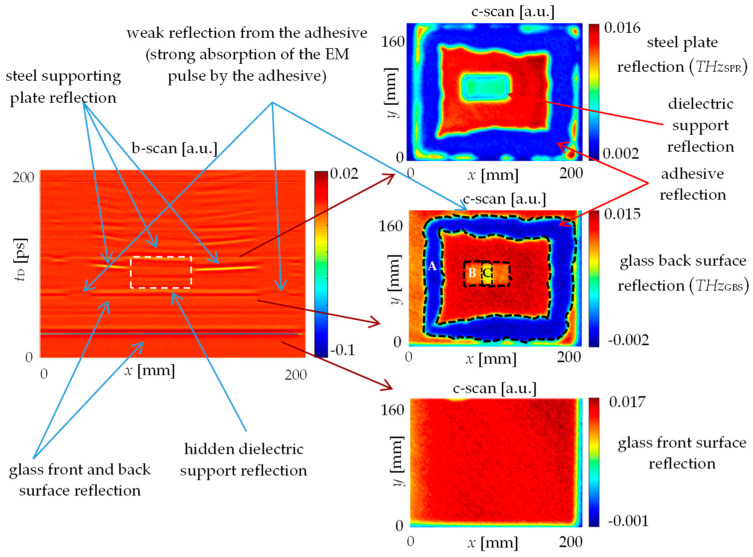
Signals obtained during pulsed terahertz inspection: b-scan (**left** side) and c-scans measured in case of various depths (**right** side); (A)—adhesive path, (B)—dielectric support, (C)—glass–support adhesive connection.

As was mentioned earlier, terahertz imaging, due to short wavelengths and enough penetrability for dielectric structures, enables one to obtain very good and reliable results. The exemplary results of pulsed terahertz inspection in the form of b-scans and c-scans are presented in Figure 10.

The b-scan represents the cross-sectional profile of the examined structure. One can observe clear pulses caused by reflections from the glass and the steel boundaries. It is also possible to see that the steel plate was not exactly planar. In the c-scans (spatial signal distributions in the case of the selected time delay, which can be recalculated to depth), the reflections from various interfaces were shown:

- the glass front surface reflection (the air–glass interface)—parameter *THz_GSR_*;
- the glass back surface reflection (the glass–air and the glass–adhesive interface);
- the steel plate reflection (the air–steel and the adhesive–steel interface)—parameter *THz_SPR_*.

In the case of the back surface of the glass slab, there was a clear indication of adhesive footprint.

Because of more similar refractive indices values of the glass and the adhesive, as well as the strong absorption of terahertz frequency electromagnetic waves by the adhesive material, the pulse reflection of the glass–adhesive interface had a much smaller amplitude compared to the glass–air interface. Lack of adhesive defect type was easily detectable. In the case of lack of adhesion to the glass, there would be very thin layer of air between the glass and the adhesive, and thus reflection in this case would be increased, causing detection possibility. For the same reason, the pulse reflected from the adhesive–steel interface was not detectable (the electromagnetic wave attenuation in the lossy adhesive after passing twice its thicknesses caused reduction of the pulse to the level lower than the noise level). Thus, in cases of such high attenuation, it is possible only to detect lack of adhesive type defect. Lack of the adhesion to the steel is not possible to detect.

Generally, after terahertz inspection of the examined adhesive path in the glass–steel joint, no lack of adhesive nor lack of adhesion defects were found. In the case of the steel plate reflection c-scan, the footprint of the adhesive was more blurred (less defined boundaries) and the thickness of the adhesive path was larger compared to the glass back surface reflection c-scan. This effect was caused by scattering of electromagnetic waves in the glass slab, defocusing of the terahertz beam after passing the focal point (in the case of higher depths) and nonrectangular geometry of the adhesive trace cross-section.

Based on the results, in the center of the sample, the dielectric support (a glass-supporting element) was traced. Its rectangular footprint was visible in c-scans because of the increased delay caused by the lower electromagnetic wave velocity (*v*_p_ = 2.04 × 10^8^ m/s) in dielectric material other than vacuum/air. The averaged refractive index (in terahertz frequency range) of support was *n*_s_ = 1.47 (which corresponds to permittivity *ε*_rs_ = 2.16).

### 3.2. Microwave Imaging

**Figure 11:**
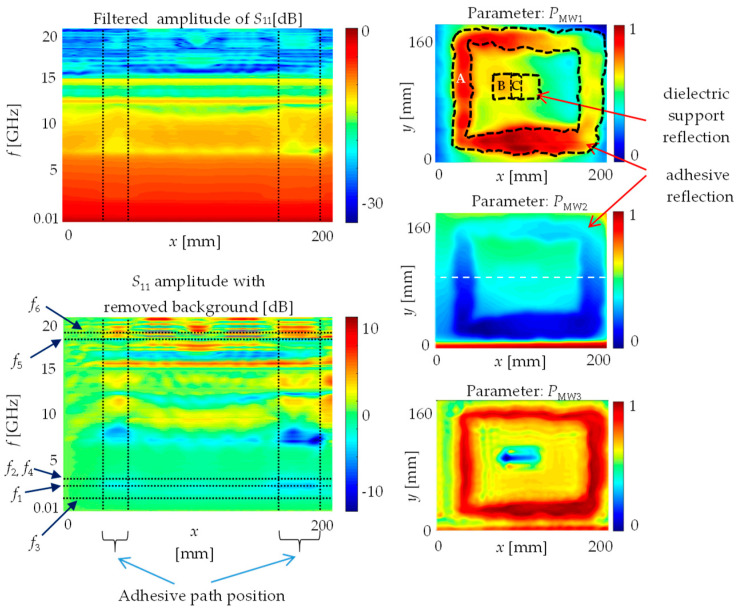
Signals obtained during microwave inspection: spatial spectrograms (**left** side) and two-dimensional distributions of selected parameters normalized to the 〈0;1〉 range (**right** side). Parameters: *P*_MW1_—integral of amplitude–frequency response for selected frequency band 〈*f*_1_; *f*_2_〉. *P*_MW2_—integral of phase–frequency response for selected low frequency band 〈*f*_3_; *f*_4_〉. *P*_MW3_—integral of phase–frequency response for selected high frequency band 〈*f*_5_; *f*_6_〉. A white dashed line marks the line along which the spatial spectrograms were measured. (A)—adhesive path, (B)—dielectric support, (C)—glass–support adhesive joint.

The measurements carried out using the microwave imaging system were made in the frequency domain (frequency sweep of VNA). The exemplary results are shown in Figure 11 (left column) in the form of spatial spectrograms and selected parameter distributions.

The spatial spectrogram shows the reflection coefficient S_11_ frequency content for various positions of the coaxial sensor after the linear (1D) scan over the sample. One could observe the frequency content changed with the sensor position. In the case of some specific frequency bands, the amplitude of *S*_11_ was increasing or decreasing in the vicinity of the adhesive path position. Such bands were identified based on the spatial spectrogram, and then the three parameters were proposed and calculated in these bands:

- *P*_MW1_—integral of amplitude–frequency response for selected frequency band, Equation (1)
  (1) $$P_{\mathrm{MW}1}\left(x,y\right)=\int_{f_{1}}^{f_{2}}\left|S_{11}\left(f,x,y\right)\right|\;df$$
- *P*_MW2_—integral of phase–frequency response for selected low frequency band, Equation (2)
  (2) $$P_{\mathrm{MW}2}\left(x,y\right)=\int_{f_{3}}^{f_{4}}\arg\left[S_{11}\left(f,x,y\right)\right]\;df$$
- *P*_MW3_—integral of phase–frequency response for selected high frequency band, Equation (3)
  (3) $$P_{\mathrm{MW}3}\left(x,y\right)=\int_{f_{5}}^{f_{6}}\arg\left[S_{11}\left(f,x,y\right)\right]\;df$$
  where, *f*—frequency; *f*_n_—cutoff frequencies in the case of selected bands: *f*_1_ = 2.79 GHz, *f*_2_ = 3.19 GHz, *f*_3_ = 1.79 GHz, *f*_4_ = 3.19 GHz, *f*_5_ = 17.98 GHz, *f*_6_ = 18.38 GHz.

The two-dimensional distribution of the proposed parameters is presented in Figure 11 (right column).

One can observe similar indications as in the case of the terahertz inspection. The adhesive path position was clearly detected, contrary to the dielectric support indication, which was less visible and its rectangular geometry was not correctly mapped. No defects of lack of adhesive or lack of adhesion were detected. Moreover, one could see additional signals not caused by the existence of adhesive between the steel and the glass layers. Those signals were caused by the edge effect, variation of liftoff and geometry of coaxial probe.

A relatively good and unambiguous result can be obtained for quite low frequency values (e.g., 3.2 GHz). This means that if we do not care about obtaining depth information (which is available in the case of the terahertz method with pulsed excitation), it is possible to develop a measurement system with a much lower cost due to the relatively low prices of vector network analyzers for the 3 GHz band.

### 3.3. Thermographic Imaging

**Figure 12:**
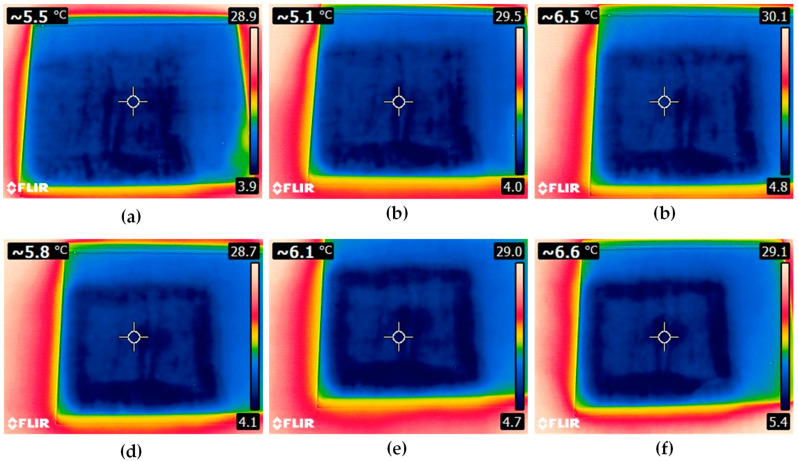
View of the sample: (**a**) at the beginning of temperature equilibration process, (**b**) 30 s at 30 °C, (**c**) 60 s at 30 °C, (**d**) 90 s at 30° C, (**e**) 120 s at 30 °C, (**f**) 150 s at 30 °C.

Selected results in the form of temperature distribution are shown in Figure 12.

**Figure 13:**
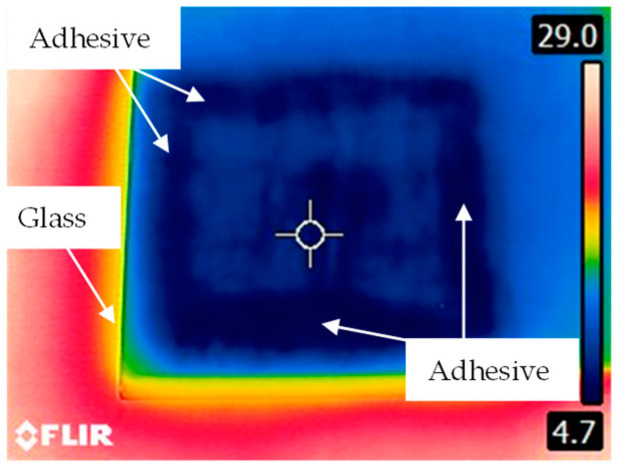
View of the sample with visible and indicated areas of adhesive occurrence in combination—glass side examination.

In the places where the adhesive was applied, the traces are depicted with different colors than in other case (Figure 13).

As mentioned earlier, the differences in the thermal conductivity of individual materials making up the structure of the sample influenced the dynamics of the temperature equilibration process. Each subsequent image of Figure 12 shows a clearly higher temperature of the steel, which is related to the faster course of the process for this material. From the point of view of joint assessment, observation of distribution recorded on the glass surface is much more important. Analyzing the time sequence of the recorded images (Figure 12a–f) one can see a gradually increasing contrast between individual areas on the sample surface. Darker shades of blue correspond to the places where there was a layer of the adhesive in the setup between the steel and glass (see Figure 13). Probably during the cooling process, the adhesive layer reached a lower temperature value than the air inside the sample. In areas where the glass contacted the adhesive layer, the equilibration process was slower, and a greater temperature contrast appeared. In Figure 12, in addition to a clear rectangular-shaped darker frame indicating the course of the adhesive path, one can also see a rectangular solid object (the glass support) in the middle of the inner area. This was also observed in the results of both previously presented electromagnetic methods.

### 3.4. Ultrasonic Imaging

**Figure 14:**
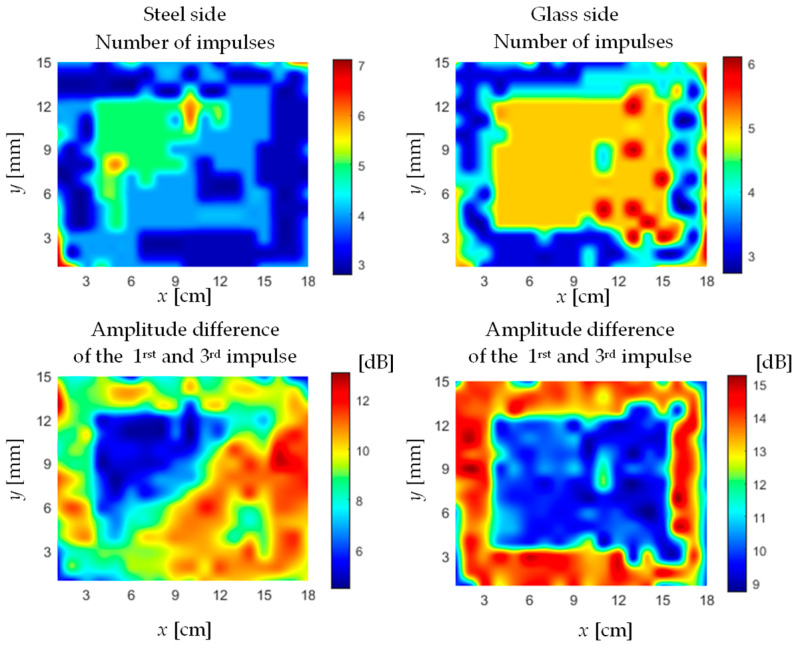
Ultrasonic test results: number of impulses—test from steel (**left** side); number of impulses—test from glass (**right** side); the difference in the amplitude between the first and the third impulse (*UT*_S_)—test from the steel (**left** side); the difference in the amplitude between the first and the third impulse (*UT*_G_)—test from the glass (**right** side).

The results of the ultrasonic tests are presented in the form of surface charts (Figure 14). These charts illustrate the distribution of selected longitudinal ultrasonic wave parameters over the entire measuring area from the steel and the glass sides.

**Figure 15:**
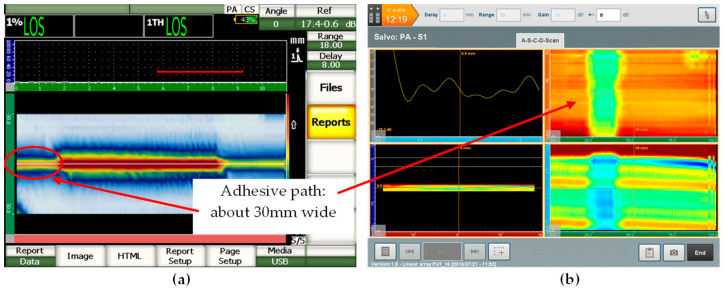
Results of ultrasonic test: (**a**) c-scan, (**b**) surface scan.

Results achieved using the UT phased array technique allowed us to visualize the adhesive path for the combination of the glass and the steel elements. Selected results for this study are shown in Figure 15.

The ultrasonic measurements confirmed that the adhesive was applied on the entire boundary of the glass plate. The adhesive path width was about 30 mm. Due to the different acoustic properties of the tested materials (steel, adhesive, glass) different ultrasonic measures were adopted. The test from the glass side *UT*_G_ confirmed that the occurrence and width of the adhesive path was best visible based on the difference in the amplitude between the first and the third impulse reflected from the joint area. The test results—number of impulses from the bond area—regardless of which side the test was carried out showed different adhesion of the adhesive to the steel substrate and the glass. The ultrasonic examination using the phased array technique confirmed the presence of the adhesive path in the joint and enabled its graphical representation.

### 3.5. Multi-Source Data Compilation

**Figure 16:**
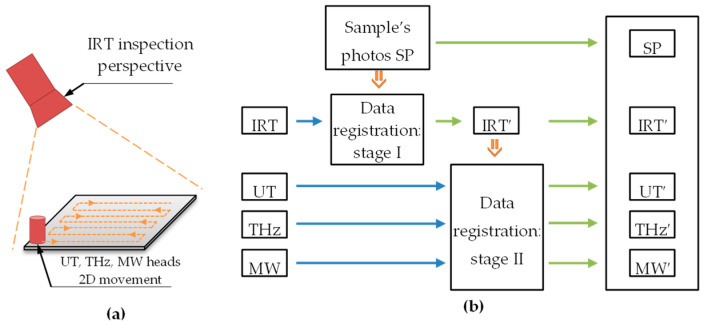
Schematic of the multiple inspections: (**a**) measurements setup, (**b**) diagram of multiple data compilation.

The operation of all methods was included in the multi-source inspection system, the purpose of which was to combine data into a common result, which made it possible to more accurately determine the location of the adhesive path. At the same time, multi-source data application allowed us to determine and verify the information carried out by the individual methods in the context of competitiveness or complementarity. A diagram of a multi-module measuring system together with a block diagram illustrating the procedure of cooperation of individual methods is presented in Figure 16.

Each of the sample inspection methods provided imaging results in a different form, affected by geometric distortions resulting from the mutual position of the tested sample and the measuring system (Figure 16a). In the case of the infrared thermography inspection, due to the method of data acquisition (the camera placed on the side of the sample at an acute angle to its surface), the resulting image represents a perspective projection of the measurement sample. However, the heads of the other measuring systems moved directly on the surface of the tested sample. It should also be emphasized that each method, due to various measuring steps, provides images of the areas of interest with different spatial resolutions. A detailed summary of the technical parameters of the results obtained from the multi-source system is shown in Table 1.

In order to enable comparison of the obtained multi-source images and the integration of their information, it was necessary to carry out the spatial registration stage of all measurement data.

Due to the multiple sources of data differing both in technical parameters and the physical basis of imaging, an important factor in the correct implementation of the registration procedure is the reference image. For this purpose, scans of both surfaces of the tested sample were used to obtain images without typical photographic distortions. The whole process of determining a common measurement space for results was divided into two stages. A common element of both stages was the result obtained during the infrared thermography inspection, which enabled visualization of both the outer edges of the sample (used in the first stage) and visualization of the internal parts of the adhesive paths (used in the second stage). In the first part, the IRT image (single frame obtained after 120 s of the equilibrium process) was transformed into a coordinate system determined by the glass surface scan, after which the obtained IRT’ image was free of prospective distortion, and its resolution corresponded to the resolution of the sample’s scan. In the second part, IRT’ was used as the reference image and enabled the transformation of the remaining measurement results (UT, THz, MW), also into the space designated by the sample’s scans. During each stage of the registration process, control (characteristic) points were first determined, and then the mutual relations of the corresponding pairs of points from both images were determined. In the next step, the parameters of the mathematical model of the transformation corresponding to the distortion characteristics of a given measurement result (in relation to the reference image) were determined (see Table 1). At the end of the data transformation procedure, the resolution of the resulting images was unified by implementing data resampling. In the first stage of registration, the characteristic points corresponded to the points determined by the intersection of the edge of the sample. In the second part, the characteristic points were defined in the inner corners of the adhesive path. Before composing the images, they were also normalized, enabling a common scale.

**Figure 17:**
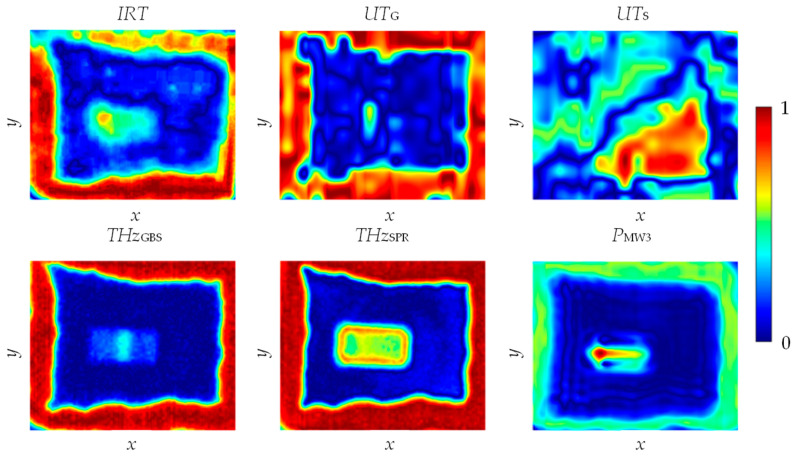
Selected multiple inspection results after the data registration process.

Selected result distributions obtained after both stages of data registration are shown in Figure 17.

As can be seen, all methods allowed assessment of the adhesive path location from the glass side. Each of the methods also indicated the presence of the additional object in the middle of the region of interest (the adhesive area of both layers). The infrared thermography method in the utilized configuration enabled volumetric inspection, and its image contained information from various layers of the tested material. In the case of the microwave method, the accurate determination of the layer depth from which the visualized information originated was also not possible. On the other hand, both the ultrasonic and terahertz methods, due to the detections techniques based on the study of wave reflection pulses (acoustic and electromagnetic, respectively) provided the opportunity to more accurately estimate the depth from which the visualized information came. Analyzing the results of *UT*_G_ and *THz*_GBS_ (which refers to the glass–adhesive interface) one could notice a clear trace of the object placed vertically in direct contact with the glass inner surface (inside the adhesive area of both layers of the composite). In deeper layers, a horizontal object was visible. This was confirmed by both the *THz*_SPR_ parameter result, illustrating reflection from the steel plate surface, and the results of other measuring methods. The result obtained for the ultrasonic method in the case of the test from the steel side confirmed that the transfer of information about the presence of the adhesive path was much less effective than in the case of the inspection from the glass side. The result provided ambiguous data, difficult to properly interpret, and therefore was not included in the further analysis.

**Figure 18:**
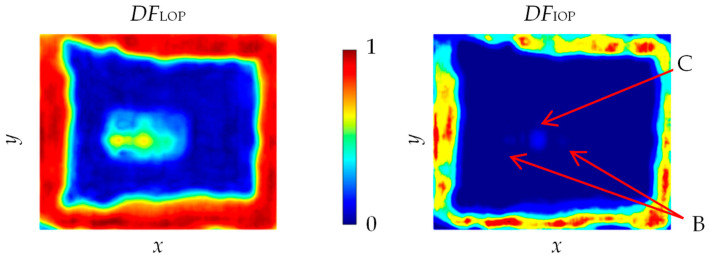
Multi-source data (of *IRT*, *UT*_G_, *THz*_GBS_, *THz*_SPR_ and *P*_MW3_) integration results under two strategies: linear opinion pooling *DF*_LOP_ and independent opinion pooling *DF*_IOP_; B—indication of dielectric support; C—indication of glass–support adhesive joint.

Next, in order to combine information provided by multiple sources, low-level fusion of data brought by all methods was carried out in two approaches. The first used two strategies for accumulating information: linear opinion pooling (LOP), and independent opinion pooling (IOP). Both are based on the theory of combining sets and use logical operators [2,3,4]. The results of applying both strategies are shown in Figure 18.

In the case of LOP (*DF*_LOP_), all information contained in the component images was reflected in the resulting image. When information was contained in a larger number of component images, it was more strongly indicated in the resulting image. This method of data fusion allows one to increase the visibility of common aspects, but also to preserve unique information of a given imaging technique. Consequently, it allows one to build a more complete result. At the same time, various types of artifacts are transformed into the resulting image in the same way, resulting from possible disturbances or disadvantages of the measuring technique. The indicated areas that were present in most of the components were particularly visible in the obtained result of *DF*_LOP_. This could be seen especially on the basis of the support object in the center of the internal area of the sample, for which the indication value increased, especially in the two centrally-crossing lines. Nevertheless, both the adhesive path and the support were clearly visible in the fused image. On the other hand, usage of the IOP resulted in strengthening common information and suppressing divergent information (*DF*_IOP_). In this way, only information that appeared in all the component images appeared in the resulting image, and unique ones were completely suppressed. Once again, one could notice the properties of this strategy especially for the support area indication (in the middle of the inner area depicted with letters B—in the location of the dielectric support and C—in the location of the glass–support adhesive), as only a single spot (lighter spot than the surrounding area but of very low intensity) in the center of the object was depicted (common part for all component images). In the case of observation of the adhesive path, one could clearly distinguish places with different intensities of indications. This confirmed differences in the adhesive path visualization in individual results, but bearing in mind that the adhesive layer was not infinitely thin and some of imaging techniques could transfer information from different depths to the distributions, the change in intensity did not carry vital information.

**Figure 19:**
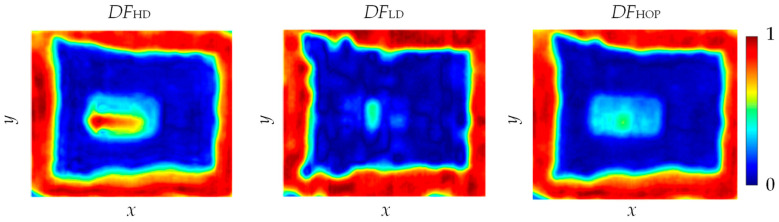
Multi-source data integration results obtained for higher depths (*DF*_HD_) and lower depths (*DF*_LD_) and hybrid opinion pooling *DF*_HOP_.

In the second approach of the data fusion procedure, a mixed—hybrid opinion pooling (HOP) strategy was used. The possibility of imaging objects at different depths from the glass side was used. Therefore, two subgroups of results were first separated, the one enabling obtaining information from the glass–adhesive interface (*THz*_GBS_ and *UT*_G_:*DF*_LD_), and another containing global information (*IRT*, *THz*_SPR_ and *P*_MW3_:*DF*_HD_). The opinions were then combined using the LOP strategy in two separate subgroups. The final result was obtained by combining the results of both subgroups using the IOP strategy (*DF*_HOP_). The results for both subgroups and the final one are presented in Figure 19.

The use of two subgroups allows for the enhancement of the joint condition information at different depths, which can be useful especially in the case of thicker adhesive layers. In the case of depicting the layers at the glass–adhesive interface (*DF*_LD_), a large variation in the width of the path was clearly visible. One could also notice a vertical mark in the place of the support, which may indicate an occurrence of the adhesive or material sticking the support to the glass. In the case of deeper layers (*DF*_HD_), one could see greater uniformity in the thickness of the path, as well as a change in shape in the center of the tested area, which corresponded to the shape of the support. The combination of the two results (*DF*_HOP_) made it possible to keep all information and to highlight to the greatest extent those which were confirmed in all the component images.

## 4. Conclusions

The aim of this paper was to investigate a selected set of methods based on different physical bases for an adhesive path inspection of a glass–steel joint on a common exemplary sample and under the same conditions. This type of joint is frequently utilized in production of various machine and vehicles and stands for the strong need for the introduction of efficient testing procedures. The conducted research allows one to evaluate the application possibilities and limitations and constitute the basis for future proper system integration. In addition, as part of the work, an attempt was also made to determine and verify the nature of information carried by individual methods in the context of their competitiveness or complementarity. The possibility and the scope of the selected testing methods are summarized in Table 2. 

In the case of methods based on high frequency electromagnetic waves (microwave and terahertz), inspection was possible only from the glass side. The glass plate enabled penetration of waves, which is clearly visible in the b-scan signal (shown in Figure 10). The thickness of the glass and the air gap between the glass and the steel plate is possible to determine. The permittivity of the adhesive is similar to the permittivity of the glass, because the amplitude of the glass–adhesive reflection pulse is noticeably smaller compared to the glass–air reflection pulse. Moreover, the utilized adhesive (similarly to many other adhesives) is lossy (imaginary part of permittivity is high), because no adhesive–steel plate reflection pulse was recorded. Pulsed excitation and time domain measurement in the case of the THz spectroscopy enable intuitive (similar to radar) analysis of obtained signals and identification of the internal state of the examined object. This method provides depth/cross-sectional information about the tested material. In the case of microwave imaging, measurement is performed in the frequency domain, and the wavelength is noticeably bigger than joint details, which are expected to be found. Thanks to the near field measurement and application of spatial spectrograms (shown in Figure 11), some parameters were proposed that enable one to obtain valuable information about the internal structure of the examined object. Comparing the results obtained with both methods using high-frequency electromagnetic waves, a large similarity can be seen. The terahertz method allows one to obtain higher resolution and depth information, while the microwave method is cheaper and works better with thicker and more lossy structures (due to deeper penetration resulting from lower frequency and relatively low power obtained in the TDS technique). The signal analysis is more complicated in the case of microwave imaging.

Inspection by the ultrasonic method both from the steel and the glass side was performed. The glass has a much smaller damping effect on propagating the ultrasonic wave, which resulted in much less amplification of the longitudinal ultrasonic wave pulse sent to the adhesive joint area. During the test, two ultrasonic parameters from a certain group were chosen: the number of ultrasonic longitudinal wave impulses, and the difference in the amplitude between the first and the third impulse obtained during testing of the joint area. These parameters were chosen because they showed best diagnostic properties in terms of the adhesive path location in the bond. The sensitivity to the presence of the adhesive path of these two diagnostic parameters is much higher for the examination from the glass side than from the steel side (shown in Figure 14). 

In order to complete the ultrasonic tests using the a-scan image, modern ultrasonic techniques with the application of a surface scanning test using a multi-transducer head were performed. Such kinds of test allow one to obtain an image of the adhesive path and measure its width both from the side of the steel and from the glass side.

The thermographic imaging method allows one to locate the adhesive in the steel–adhesive–glass joint from the side of the glass on account of the differences in the thermal conductivity of individual materials making up the structure of the adhesive joint. Reduction of the temperature of the sample to the level of about 3 °C and then leaving it at a temperature nearly 10 times higher results in the temperature equilibration process. This process can be tracked on images acquired by a IR camera (Figure 12). However, the temperature change in the tested joint is not always favorable and may cause its damage (heating/cooling the joint area beyond limits in order to observe the temperature equilibration process).

Despite the use of only one sample, the proposed methods showed high repeatability of the test. This can be stated on the basis of the large number of taken measurements and the predictable and simple shape of the joint surfaces. The application of this type of joint (flat glass–adhesive–steel) is broad and covers the automotive (connecting a glass with a frame structure in buses), the tram (a connecting glass with a vehicle structure) as well as the rail (a connecting glass with a train body) markets. From the point of view of systems and developed measurement methods, the repeatability of measurements is high. It is far more important to ensure repeatability of measuring conditions, e.g., lift-off stability (in non-contact methods) or control of the continuity of coupling of the measuring heads with the material (in contact methods), which can be ensured by robotization and calibration of measuring stations, which is currently standard in industry.

The use of multiple testing methods creates an opportunity to illustrate many different aspects of the tested joint. It is particularly interesting to use many methods based on different physical bases, thanks to which it is possible to obtain a wider spectrum of new and unique information, and thus it is possible to build a more complete knowledge. The obtained results confirmed the potential for building multi-source inspection systems. On the basis of data fusion outcomes, it can be noticed that the applied methods allow one to obtain both convergent as well as complementary information. Thus, the appropriate combination of information enables the building of a result in which both the common and the unique data are emphasized. However, for a more detailed analysis, it seems justified to have a multi-level data combination, which enables the transfer of data on the structure state at various depths. Such research will be carried out in subsequent stages of work and presented in the next paper of the authors.

## Figures and Tables

**Table 1:** Technical parameters of the inspection data.

Inspection Technique	Inspection Surface	Transformation Perspective	Inspection Area/Resolution	Reference Source
Infrared thermography	glass	projective	620 × 480 pixels	Photography of the inspected surface
Ultrasonic testing	both	similarity	150 × 180 mm/10 mm	Transformed infrared thermography image IRT’
Terahertz testing	glass	similarity	156 × 196 mm/2 mm	Transformed infrared thermography image IRT’
Microwave testing	glass	similarity	212 × 202 mm/2 mm	Transformed infrared thermography image IRT’

**Table 2:** Comparison of the individual methods.

Parameter	UT	IRT	MW	THz
Frequency test	20 MHz	>10 THz	10 MHz–20 GHz	0.1–4 THz
Glass side examination	+	+	+	+
Steel side examination	+	+	-	-
Contact/contactless	Contact	Contactless	Contactless	Contactless
Advantages of each method—test of glass–adhesive–steel joint	Test time and online result. Tests in various weather conditions.	Test time and online result.	Acceptable power level enabling examination of structures of various thicknesses.	Easily available information on the depth of the defect (time domain measurement). Relatively short wavelength allowing small defects detection.
Disadvantages of each method—test of glass–adhesive–steel joint	High damping of adhesive—the need to test from two sides. The thickness of the tested connection (joined materials) determines the choice of the ultrasonic wave frequency.	The need to change the temperature of the element during the test.	Measuring head needs to be scanned over the sample—slow process. Hardly available information on the depth of the defect (frequency domain measurement). Relatively long wavelength limiting size of detected details.	Measuring head needs to be scanned over the sample—slow process Low power limiting the thickness of examined dielectric structures.
Feedback parameters from joint area	Amplitude of several impulses. Number of impulses.	View of temperature distribution.	Amplitude or phase of scattering matrix parameter *S*_11_ (reflection coefficient). Parameters calculated based on integrals of *S*_11_ over specific frequency bands.	Distribution of time domain signal associated with specific delay (correlated with depth)—c-scan signal.
